# Diabetes Mellitus and Mortality after Acute Coronary Syndrome as a First or Recurrent Cardiovascular Event

**DOI:** 10.1371/journal.pone.0003483

**Published:** 2008-10-22

**Authors:** Richard M. Cubbon, Afroze Abbas, Stephen B. Wheatcroft, Niamh Kilcullen, Raj Das, Christine Morrell, Julian H. Barth, Mark T. Kearney, Alistair S. Hall, for the EMMACE-2 investigators

**Affiliations:** 1 Division of Cardiovascular and Diabetes Research, Leeds Institute of Genetics, Health & Therapeutics, The LIGHT Laboratories, The University of Leeds, Leeds, United Kingdom; 2 C-NET Group, Clinical Cardiology, Leeds General Infirmary, Leeds, United Kingdom; 3 Department of Clinical Biochemistry and Immunology, Leeds General Infirmary, Leeds, United Kingdom; L' Istituto di Biomedicina ed Immunologia Molecolare, Consiglio Nazionale delle Ricerche, Italy

## Abstract

**Background:**

Diabetes Mellitus (DM) is associated with adverse cardiovascular prognosis. However, the risk associated with DM may vary between individuals according to their overall cardiovascular risk burden. Therefore, we aimed to determine whether DM is associated with poor outcome in patients presenting with Acute Coronary Syndrome (ACS) according to the index episode being a first or recurrent cardiovascular event.

**Methods and Findings:**

We conducted a retrospective analysis of a prospective cohort study involving 2499 consecutively admitted patients with confirmed ACS in 11 UK hospitals during 2003. Usual care was provided for all participants. Demographic factors, co-morbidity and treatment (during admission and at discharge) factors were recorded. The primary outcome was all cause mortality (median 2 year follow up), compared for cohorts with and without DM according to their prior cardiovascular disease (CVD) disease status. Adjusted analyses were performed with Cox proportional hazards regression analysis. Within the entire cohort, DM was associated with an unadjusted 45% increase in mortality. However, in patients free of a history of CVD, mortality of those with and without DM was similar (18.8% and 19.7% respectively; p = 0.74). In the group with CVD, mortality of patients with DM was significantly higher than those without DM (46.7% and 33.2% respectively; p<0.001). The age and sex adjusted interaction between DM and CVD in predicting mortality was highly significant (p = 0.002) and persisted after accounting for comorbidities and treatment factors (p = 0.006). Of patients free of CVD, DM was associated with smaller elevation of Troponin I (p<0.001). However in patients with pre-existing CVD Troponin I was similar (p = 0.992).

**Conclusions:**

DM is only associated with worse outcome after ACS in patients with a pre-existing history of CVD. Differences in the severity of myocyte necrosis may account for this. Further investigation is required, though our findings suggest that aggressive primary prevention of CVD in patients with DM may have beneficially modified their first presentation with (and mortality after) ACS.

## Introduction

Diabetes Mellitus (DM) is widely acknowledged to increase the risk of developing atherosclerosis in addition to doubling risk of cardiovascular death [1]. Of particular relevance, Haffner *et al* demonstrated that patients with DM, and no prior myocardial infarction (MI) suffered future MI at a rate equal to non-diabetic patients with a history of MI [2], a group warranting aggressive *secondary* preventative therapy. This underlies guidance that the presence of DM alone, in individuals free of overt cardiovascular disease (CVD), warrants the use of similarly aggressive *primary* prevention strategies [3], [4]. Furthermore, the OASIS investigators demonstrated that DM conferred added risk of cardiovascular mortality after unstable angina or non-Q wave MI in patients with or without a prior history CVD [5]. However, more recent work has contradicted these findings [6], [7]. Some of this data has shown that the cardiovascular risk attributable to DM is heterogeneous and dependent on the overall burden of cardiovascular risk factors in individual patients [7]. Hence, one might expect that the aggressive risk reduction measures now targeted at patients with DM and no prior CVD makes the mortality risk attributable to DM differ between patients with first or recurrent cardiovascular events. Furthermore, improved screening for DM may have resulted in earlier diagnosis of the disorder, potentially reducing the CV risk of current trial cohorts with DM, when compared with historical groups, such as Haffner *et al's*. In order to investigate this hypothesis we conducted an analysis of observational data pertaining to a contemporary cohort of acute coronary syndrome (ACS) sufferers.

## Methods

### Data collection

A retrospective analysis of the EMMACE-II observational cohort study was performed [8]. This examined outcomes in 2499 consecutively admitted, unselected patients with the diagnosis of ACS confirmed by cardiologists; specifically, at least two of ischaemic symptoms, new ECG features compatible with ischaemia, and biomarker elevation (cardiac troponin concentration above the 10% CV taken 12–24 hours after the onset of symptoms or raised CK concentration above twice the upper limit of normal) were required. Data was collected from 11 hospitals in West Yorkshire, UK between 28^th^ April and 28^th^ October 2003. All patients provided written informed consent and the study was conducted with appropriate local and regional ethics committee approval in accordance with the declaration of Helsinki. Potential participants were identified using a comprehensive search of clinical coding data, coronary care registers and biochemistry laboratory cardiac biomarker results. Detailed data on patient demographics, medical history, index event characteristics and management were collected and all cause mortality data (median 2 years) was provided by the United Kingdom Office for National Statistics.

Individuals with DM were identified on the basis of past history documented in the medical records, or the receipt of DM-related dietary or pharmacologic intervention prior to the index event. History of CVD was defined by the presence of any prior myocardial infarction, angina, cerebrovascular event, peripheral vascular disease or coronary revascularisation procedure. Patient age, heart rate and systolic blood pressure data were collected immediately on hospital admission. Chronic renal impairment refers to estimated glomerular filtration rate <30 ml/min/1.73 m^2^ (Cockroft-Gault method) and heart failure to any previous diagnosis. Killip class (grades 1 to 4 indicating increasingly severe signs of heart failure) pertains to the highest recording during admission. Troponin I (TnI) data was collected using the Beckman Coulter AccuTnI assay. Revascularisation refers to percutaneous coronary intervention (PCI) or coronary artery bypass grafting performed during the inpatient or early post discharge phase; reperfusion refers to use of thrombolysis or primary PCI. Secondary preventative pharmacotherapy use was defined at hospital discharge.

### Statistical analysis

All statistical analyses were performed using SPSS version 13.0 (SPSS Inc., Chicago, Illinois, USA). Continuous data are presented as mean (standard error) and categorical data as number (percentage). Groups were compared using Student's T-test or Mann-Whitney tests (non-normally distributed data) for continuous data and Pearson χ^2^ for categorical data using two-sided tests. Crude group survival data were compared using log rank tests. Statistical significance was accepted at p<0.05, though when interpreting the multiple comparisons of cohort characteristics displayed in Table 1 a value of p<0.0028 should be used (Bonferroni correction). Cox proportional hazards analysis was used to determine the significance of interaction between DM and CVD in predicting survival; the interaction term was additionally corrected for demographic and clinical variables as outlined later. Covariates were selected prior to analysis on the basis of clinical relevance to outcome; no stepwise removal was used during analysis. Missing data regarding DM or CVD status resulted in the exclusion of 72 patients (2.9%) from the analysis.

**Table 1:** Cohort characteristics.

	No CVD		p value	CVD		p value
	No DM	DM		No DM	DM	
	n = 950	n = 117		n = 1060	n = 300	
Deaths	187 (19.7)	22 (18.8)	0.74*	352 (33.2)	140 (46.7)	<0.001*
Age (years)	65.7 (0.5)	67.6 (1.2)	0.192	73.8 (0.4)	72.7 (0.6)	<0.001
Gender (male)	65.3 (620)	65 (76)	0.95	60 (636)	56 (168)	0.213
Cigarette smoking	39.4 (374)	9.4 (11)	<0.001	19.8 (210)	11(33)	<0.001
Chronic Renal Impairment	1.1 (10)	7.7 (9)	<0.001	6.3 (66)	8.1 (24)	0.271
Heart Failure	2.6 (25)	3.4 (4)	0.63	10.1 (106)	12.5 (37)	0.234
Systolic BP (mmHg)	142.9 (1.0)	144.5 (2.6)	0.338	140.6 (0.9)	143.1 (1.8)	0.824
Heart Rate (bpm)	82.5 (0.7)	84.2 (2.2)	0.82	83.4 (0.8)	90.8 (1.3)	0.234
Random Glucose (mmol/l)	7.5 (0.1)	12 (0.5)	<0.001	7.3 (0.1)	12.1 (0.4)	<0.001
Troponin I (ng/ml)	15.7 (0.9)	9.5 (1.7)	<0.001	7 (0.6)	7.4 (1.0)	0.992
Creatinine Kinase (U/l)	885 (50)	552 (78)	<0.001	451 (34)	413 (50)	0.447
Killip Class	1.26 (0.02)	1.35 (0.06)	0.23**	1.39 (0.02)	1.46 (0.04)	0.065**
ST elevation	39.5 (374)	29.1 (34)	0.029	18.1 (191)	15.2 (45)	0.234
Revascularisation	19.8 (187)	21.4 (25)	0.695	15.8 (166)	11 (33)	0.038
Reperfusion	31.9 (303)	24.8 (29)	0.115	10.8 (114)	11 (33)	0.898
Aspirin	80.6 (752)	79.5 (93)	0.775	72.5 (754)	68.7 (206)	0.194
Statin	79.2 (738)	85.2 (98)	0.128	73.6 (767)	76.1 (220)	0.387
ACE inhibitor	60.9 (573)	71.6 (83)	0.026	56.6 (589)	62.7 (183)	0.062
Beta-blocker	69.5 (648)	70.2 (80)	0.887	55.9 (580)	51.2 (149)	0.157
Clopidogrel	36 (341)	38.5 (45)	0.608	40.3 (425)	45.3 (135)	0.123

* Log-rank test.

** Mann-Whitney test.

BP = Blood Pressure.

## Results

### Crude mortality data

**Figure 1:**
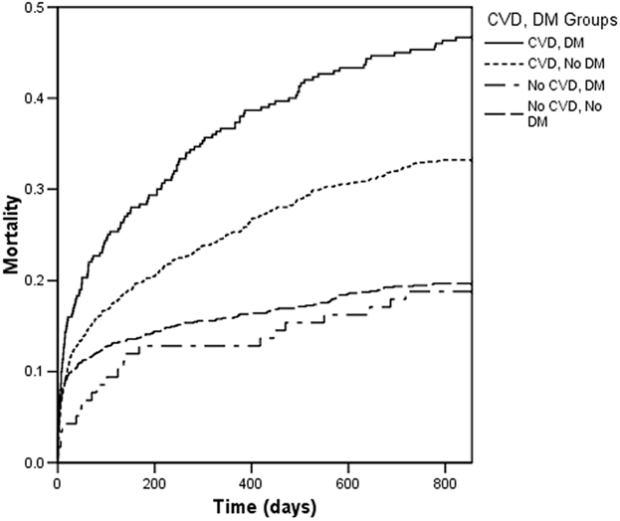
Cohort mortality. Kaplan-Meier curves illustrating mortality of the 4 study cohorts according to pre-existing CVD and DM status.

Within the entire cohort 17.2% of patients were known to suffer from DM and 58% had established CVD. The crude mortality of patients with and without DM was 38.8% and 26.8%, respectively (relative risk 1.45) over 4030 patient-years of follow up. However, for the cohort without prior CVD, patients with and without DM exhibited similar mortality (18.8% and 19.7% respectively; Hazard Ratio associated with DM 0.95; p = 0.74 by log rank; Figure 1). In contrast, in the cohort with known CVD, patients with DM had a significantly higher mortality than those without DM (46.7% and 33.2% respectively; Hazard ratio associated with DM 1.41; p<0.001 by log rank). Confirming the observation that DM confers differing mortality risk according to presentation with first or recurrent ACS, the interaction between CVD and DM in predicting mortality was significant (p = 0.039).

### Cohort characteristics

The characteristics of patients with and without CVD, according to their DM status, are detailed in Table 1. In patients free of prior CVD, DM was associated with lower rates of ST-elevation MI (29.1% vs. 39.5%; p = 0.029); this contrasts with similar rates of ST elevation in patients with known CVD, independent of DM status (p = 0.234). Troponin I, an index of myocyte necrosis, was significantly lower in patients with DM and no prior CVD, compared with patients without DM (p<0.001) suggesting smaller ‘infarct size’. In contrast, the cohort with known CVD exhibited similar troponin elevations independent of DM status (p = 0.992); the interaction between DM and CVD in predicting TnI was significant (p = 0.002).

### Adjusted mortality data

After adjusting for age and gender differences between groups the interaction between DM and CVD in predicting mortality was highly significant (p = 0.002). Further adjustment for comorbidity and treatment factors outlined in Table 1 (Chronic renal impairment, heart failure, reperfusion therapy, early revascularisation; use of aspirin, clopidogrel, statins, ACE inhibitors and beta-adrenoreceptor antagonists) did not result in loss of this interaction (p<0.006). However after accounting for ‘Troponin I as a surrogate for infarct size’, the interaction between CVD and DM in predicting mortality lost statistical significance (p = 0.056) suggesting differences in ‘infarct size’ may account for some of our observations. Table 2 provides hazard ratios for the risk attributable to DM in cohorts with and without prior CVD for each of these adjusted models.

**Table 2:** Adjusted analyses.

Adjusted model	No Cardiovascular disease		Cardiovascular disease	
	HR	p value	HR	p value
Age and sex	0.83 (0.53–1.29)	0.397	1.75 (1.45–2.14)	<0.001
Above plus co-morbidity and treatment factors	0.84 (0.53–1.34)	0.464	1.69 (1.39–2.11)	<0.001
Above plus Troponin I	0.95 (0.55–1.62)	0.837	1.68 (1.32–2.13)	<0.001

Hazard ratios (with 95% confidence intervals) for the risk attributable to DM within cohorts with and without prior CVD are displayed for the 3 adjusted analyses performed. HR = Hazard Ratio.

### Alternative CVD definitions

The presented analyses defined CVD as the presence of any prior myocardial infarction, angina, cerebrovascular event, peripheral vascular disease or coronary revascularisation procedure. Importantly, our observations persist when changing the definition of CVD to 1) exclude cases defined solely on the basis of angina; 2) exclude cases defined solely on the basis of angina, or coronary revascularization (Data not shown).

## Discussion

Our study of a contemporary cohort of ACS patients suggests that the notion of DM increasing mortality risk in *all* ACS sufferers needs to be revisited. The ability to predict high risk groups after ACS is a crucial aspect of day-to-day management of individual patients, and is also important in guiding allocation of limited resources. Whilst DM is undoubtedly associated with poor outcome in entire ACS cohorts [1], we have shown that its negative prognostic value is greatest in patients with recurrent CVD, as opposed to those whose ACS is their first CVD presentation.

The reasons for these findings cannot be explained by an observational study, though the differences in ACS subtype and extent of myocyte necrosis between groups is intriguing. Indeed, the addition of TnI as an index of ‘infarct size’ to our adjusted model resulted in loss of the interaction between CVD and DM in predicting mortality, even after accounting for other demographic, comorbid and treatment factors. In other words, the smaller ‘infarct size’ of patients with DM and no prior CVD, compared to patients without DM or prior CVD, may account for their similar mortality rates.

Whilst the smaller ‘infarct size’ of patients with DM in the cohort free of prior CVD is significant, we again cannot explain this due to the observational nature of the study. However, patients with DM are known to exhibit more diffuse coronary artery disease and it may be that their vulnerable plaques are more distal [9], [10], so threatening a smaller volume of myocardium. Equally, the well documented decline in incidence of ST elevation MI [11], which is attributed to increasingly aggressive primary and secondary prevention strategies, may be relevant. Since the publication of Haffner *et al's* work a decade ago [2], aggressive primary prevention of CVD in patients with DM has become routine [3], [4]. This may have ‘stabilised’ the sub-clinical atherosclerosis of patients with DM, reducing their infarct size below that of non-diabetic patients who may have received less effective CVD prevention measures as a group. Such changes in primary prevention may also explain the differences between our study and the OASIS investigators whose cohort was studied in 1995–6 [5].

### Study limitations

A number of limitations should be borne in mind when reading the present report. The analysis would have benefited from measurements of other novel and traditional cardiovascular risk factors such as albuminuria and obesity, in addition to detailed analysis of preventative therapy received by patients prior to the index event. Future investigation as to why diabetes is associated with less mortality risk in patients free of prior CVD would benefit from their inclusion. Furthermore, rates of secondary preventative therapy provision at hospital discharge in our study appeared counterintuitive, with prior CVD sufferers less likely to receive statins and ACE inhibitors than patients suffering their first CVD event. Whether this relates to the greater age and co-morbidity of prior CVD sufferers (resulting in reluctance to prescribe in case of relative contraindications, drug interactions or side-effects) is unclear. Again further studies addressing this would be useful.

### Future studies

Further clinical trials may serve to test our observations without the inherent disadvantages of observational research. However, our work raises a number of important questions. First, if aggressive primary prevention of CVD in patients with DM has reduced their mortality to a level comparable to those without DM, should we be targeting more aggressive primary preventative management to patients without DM? Second, if it is possible to reduce the mortality of patients with DM and first ACS to a rate comparable to patients without DM, can we maintain this achievement by more aggressively preventing further CV events? Our data suggests there is capacity to improve the use of evidence based secondary preventative measures in this group. Equally, if the mortality of patients with DM and no prior CVD has been reduced to the level of patients without DM how can we mirror this in patients presenting with recurrent ACS? This is perhaps the most challenging question and may well require novel therapies in addition to aggressive adoption of currently accepted therapies. Such questions will undoubtedly become increasingly important as the prevalence of DM continues to rise.
